# Assessing Trait Emotional Intelligence in Youth: A Psychometric Evaluation of the Short Form for the Emotional Quotient Inventory Youth Version

**DOI:** 10.3390/ejihpe16060081

**Published:** 2026-06-12

**Authors:** Colin T. Henning, Emily Storey-Hurtubise, Yelnura N. Autalipova, Samantha M. Van Rens, Laura J. Summerfeldt, James D. A. Parker

**Affiliations:** 1 Department of Psychology, Trent University, 1600 West Bank Drive, Peterborough, ON K9L 0G2, Canada; colinhenning@trentu.ca (C.T.H.); samanthavanrens@trentu.ca (S.M.V.R.);; 2 Humanities Department, SDU University, Kaskelen City 040900, Kazakhstan

**Keywords:** trait emotional intelligence, children, adolescents, self-report, psychometric properties

## Abstract

The present study examined the psychometric properties of a short form of the Emotional Quotient Inventory Youth Version (EQ-i:YV-S), a 30-item scale developed to be an efficient tool to assess trait emotional intelligence (TEI) in children and adolescents. The 4-factor model of TEI used to develop the measure (also used in the development of the original adult form) consists of intrapersonal, interpersonal, adaptability, and stress management dimensions. Confirmatory factor analysis showed acceptable fit in a large sample of youth and was invariant across gender and education level. The results also showed the EQ-i:YV-S scale to have good internal and 6-month test–retest reliabilities. Relationships between the TEI measure and alexithymia in older adolescents provide additional evidence of convergent validity. Overall, the results suggested that the EQ-i:YV-S is a valid and reliable short, multidimensional measure of core TEI dimensions in youth.

## 1. Assessing Trait Emotional Intelligence in Youth: A Psychometric Evaluation of the Short Form for the Emotional Quotient Inventory Youth Version

Research on the conceptualization and measurement of multidimensional models of emotional intelligence (EI) began with the seminal work of Salovey and Mayer (1990). However, subsequent developments and debate in the field on how best to define and measure EI led Petrides and Furnham (2001) to propose a distinction between ability EI (AEI), assessed through performance-based measures of emotional knowledge and processing, and trait EI (TEI), assessed through self-reports of typical emotion-related behavior and dispositions. Alternative frameworks for categorizing EI models have subsequently been proposed, such as the three-streams of EI proposed by Caruso (2004; i.e., ability, trait, and competency models), and the three-streams proposed by Ashkanasy and Daus (2005; i.e., ability, mixed, and trait models). However, given the high correlation among self-report measures across EI models regardless of stream and their low correlations with performance-based measures (Siegling et al., 2015), this paper refers only to Trait and Ability EI as proposed by Petrides and Furnham (2001).

Accordingly, TEI refers to a collection of emotion-related competencies, dispositions, and self-perceptions involved in perceiving, understanding, expressing, and regulating one’s own emotional states and those of others (Bar-On & Parker, 2000). Though there exists a variety of conceptual models of TEI, including within youth populations, TEI measures have generally demonstrated strong associations with socioemotional functioning (Parker et al., 2017; Zeidner et al., 2012). Given this, it is not surprising that empirical studies have consistently found TEI is a crucial personal resource supporting psychological resilience and positive outcomes across multiple life domains (Ang & Lau, 2024; MacCann et al., 2020; Parker et al., 2021). For instance, in educational contexts, higher TEI is associated with higher academic performance, more positive student–teacher relationships, less bullying behavior, and greater academic motivation in youth (Droppert et al., 2019; Mavroveli & Sánchez-Ruiz, 2011; Chamizo-Nieto et al., 2021; Taibolatov et al., 2024). In the workplace, youth with higher TEI report higher employability and career decision-making self-efficacy, and better leadership skills (Di Fabio & Kenny, 2015; McElravy & Hastings, 2014). Additionally, higher TEI is correlated with various mental health outcomes and youth development (Cao & Chen, 2025; Llamas-Díaz et al., 2022; Vega et al., 2022). As a result, researchers have expanded efforts to develop psychoeducational interventions designed to improve various emotional and social competencies in youth (Durlak et al., 2022; Storey-Hurtubise et al., 2022), often to foster resilience, prevent bullying, and promote well-being (Taylor et al., 2017).

### 1.1. Measurement of TEI in Youth

Given the growing research interest in TEI over the last few decades, it is perhaps unsurprising that a variety of assessment tools have been developed. Widely used instruments designed to assess TEI are available through both the journal literature (e.g., the Self-Report Emotional Intelligence Test, SREIT, Schutte et al., 1998; and the Wong and Law Emotional Intelligence Scale, WLEIS, Wong & Law, 2002) and commercial publishers (e.g., the Bar-On Emotional Quotient Inventory, EQ-i; Bar-On, 1997; the Swinburne University Emotional Intelligence Test, SUEIT, Palmer & Stough, 2001; and the Trait Emotional Intelligence Questionnaire, TEIQue, Petrides et al., 2007). It should be noted, however, that only a small subset of these measures designed for adults has been adapted and validated for use with youth populations (Özal et al., 2024).

One of the most widely used measures of TEI adapted for use with youth is the Emotional Quotient Inventory (EQ-i; Bar-On, 1997), which conceptualizes TEI as a constellation of four broad emotional and social competencies (ESCs): Intrapersonal (perceived ability to label, understand, and express one’s own emotions); Interpersonal (perceived ability to understand and empathize with the feelings of others); Adaptability (perceived ability to appraise, problem-solve, and persevere in challenging situations); and Stress Management (perceived emotional sensitivity and ability to regulate difficult emotions). The psychometric properties of the original EQ-i, designed for use in adults, have been widely examined over the last several decades and found to be robust (Kun et al., 2012; Parker et al., 2009, 2011).

Shortly after the development of the EQ-i, to assess TEI in children and adolescents ages 7 to 18, the 60-item Emotional Quotient Inventory–Youth Version (EQ-i:YV) was adapted from the original EQ-i using the same 4-factor theoretical model of TEI (Bar-On & Parker, 2000). Subsequently, for pragmatic reasons related to the feasibility of using such a long measure in time-constrained settings (e.g., schools and clinical evaluations), the authors also developed a short form of the measure consisting of 30 items to assess the four TEI dimensions found in the EQ-i:YV (i.e., the EQ-i:YV-S; Bar-On, 1997). This short multidimensional measure of TEI in youth represented a unique assessment tool in the broader youth EI literature, as other existing TEI measures designed for youth are limited to only a global TEI scale due to psychometric shortcomings (Davis & Wigelsworth, 2018). Nonetheless, apart from the original work reported in the test manual on the development of the EQ-i:YV-S (Bar-On & Parker, 2000), little independent work has been published evaluating the psychometric properties of the short measure, particularly in English-speaking samples. Additionally, psychometric information on the short form reported in the original EQ-i:YV manual itself utilized participants who had completed the long form, rather than the EQ-i:YV-S as a standalone measure.

To date, there are only a small number of international studies that have reported on the psychometric properties of the EQ-i:YV-S (Davis & Wigelsworth, 2018; Kun et al., 2012; Navarro-Roldán et al., 2023), with none yet providing adequate psychometric information. For example, a study by Kun et al. (2012) examined the factor structure of the EQ-i:YV-S in a sample of Hungarian adolescents. However, multiple aspects of their psychometric analyses were flawed, including the removal of four items related to the interpersonal and intrapersonal scales on the measure prior to factor analyzing their data, as well as the inclusion of items from the EQ-i:YV-S’s Positive Impression Index—items not part of the measure’s TEI model. The findings of Kun et al. (2012), therefore, provide poor psychometric evidence regarding the EQ-i:YV-S, with limited generalizability to other studies utilizing the measure. More recent studies by Davis and Wigelsworth (2018), Navarro-Roldán et al. (2023), Esnaola et al. (2016), and Esnaola et al. (2018) provide a more robust examination of the psychometric properties of the EQ-i:YV-S in adolescent samples from the UK, Colombia, Spain, and China, respectively. These studies include examinations of the measure’s reliability, factor structure, convergent and discriminant validity, and predictive validity. However, these studies generally examined only internal reliabilities, and only one study to date has tested measurement invariance with respect to gender (i.e., Davis & Wigelsworth, 2018). This limits conclusions regarding the temporal stability of the EQ-i:YV-S, which purports to measure a trait construct, and the comparability of the measure’s factor structure across boys and girls. Further, to our knowledge, no study has yet examined the measurement invariance of the EQ-i:YV-S across age or education level (i.e., elementary vs. high school), a particularly problematic gap in the current literature, as the EQ-i:YV-S was designed for use in youth as young as 7 years old. Existing evidence on the convergent validity of the EQ-i:YV-S with other related constructs is also currently limited. Previous studies have only examined associations between the EQ-i:YV-S and measures of self-concept (Esnaola et al., 2016, 2018), coping (Navarro-Roldán et al., 2023), and two other measures of EI (Davis & Wigelsworth, 2018).

### 1.2. Present Study

The present study sought to address these gaps in the literature on the EQ-i:YV-S by investigating a range of psychometric properties for the measure in a large sample of children and adolescents ages 9 to 16. Specifically, the present study used EQ-i:YV-S data collected from several large groups of Canadian elementary and high school students to examine the factor structure of the EQ-i:YV-S in English-speaking youth, including gender and education level measurement invariance, as well as internal and test–retest reliabilities, and convergent validity with another measure of emotion-related competencies.

## 2. Method

### 2.1. Participants and Procedure

Data used in this study came from students attending several different elementary and high schools in the Central Ontario area in Canada: 1079 elementary school-aged children (582 boys, 497 girls) and 1067 high school adolescents (473 boys, 594 girls) who completed the short form of the EQ-i:YV. Collectively, participating youth ranged in age from 9 to 16, with a mean age of 13.39 years. A subsample of 133 elementary school students (63 boys, 70 girls) completed the EQ-i:YV-S at two different time points, approximately six months apart. Another subsample of 109 high school students (47 boys and 62 girls) completed a self-report measure of alexithymia.

### 2.2. Measures

The Emotional Quotient Inventory–Youth Version Short Form (EQ-i:YV-S) is a brief 30-item self-report measure of TEI designed to measure social and emotional competencies in children and adolescents ages 7 to 18 years old (Bar-On & Parker, 2000). Respondents are asked to rate items on a 4-point Likert scale (1 representing “very seldom or not true of me” to 4 representing “very often true or true of me”). Items are scored on the same four EI subscales as the original full-length youth version: Intrapersonal, Interpersonal, Stress Management, and Adaptability. Together, the four scales are also summed to calculate a Total TEI scale. Higher scores on each scale indicate higher levels of emotional and social competence. The EQ-i:YV-S also includes a 6-item Positive Impression Index, which is not part of the measure’s TEI model. Therefore, only the 24 items from the measures’ four TEI scales were analyzed in this study.

The 20-item Toronto Alexithymia Scale (TAS-20; Bagby et al., 1994) is a widely used self-report measure designed to assess the alexithymia construct with a 5-point Likert scale format (1 representing “strongly disagree” to 5 representing “strongly agree”). Higher scores on this measure indicate higher levels of alexithymia. The TAS-20 provides three subscales reflecting the three core facets of alexithymia: Difficulties Identifying Feelings (DIF), Difficulties Describing Feelings (DDF), and Externally Oriented Thinking (EOT). The three subscales are also summed for a Total Alexithymia scale (TAS-20). The reliability and validity of the TAS-20 have been well-established in diverse samples (Bagby et al., 2020), and scores on the TAS-20 have been found to be negatively correlated with scores on other measures of EI (Bagby et al., 2020; Davidson & Morales, 2022).

### 2.3. Statistical Procedures

***Testing Factor Structure***

All analyses were conducted in JASP version 0.19.3 (JASP Team, 2024). To test the 4-factor structure of the EQ-i:YV-S items, we used confirmatory factor analysis (CFA) and an overall evaluation strategy consistent with other similar measures of TEI (Feher et al., 2019; Van Rens et al., 2024). Diagonally Weighted Least Squares (DWLS) was the estimation method used for all CFAs. To evaluate the goodness of model fit, we used the following robust indices: the Comparative Fit Index (CFI), the Standardized Root Mean Square Residual (SRMR), and the Root Mean Square Error of Approximation (RMSEA) with a 90% confidence interval (CI). The following graded fit criteria were used to evaluate overall model fit based on previously recommended cut-off values (Marsh et al., 2004): CFI ≥ 0.95, SRMR and RMSEA ≤ 0.05 were suggestive of good model fit; CFI ≥ 0.90, and SRMR and RMSEA ≤ 0.08 for acceptable fit. Misfit in the model was further evaluated by examining individual factor loadings (e.g., expected factor loadings ≥ 0.30; Brown, 2006) and standardized residuals.

***Measurement Invariance Across Gender and Education Level***

Measurement invariance of the 4-factor structure of the EQ-i:YV-S was tested across gender (i.e., boys vs. girls) and education level (i.e., elementary school vs. high school) using a multi-group CFA framework, fitting the same model for the two groups simultaneously and imposing increasingly restrictive cross-group equality constraints on model parameters. First, we tested a baseline model with equality constraints on only the number of factors and the pattern of item-factor loadings (configural invariance). Then, we conducted a test of invariant factor loadings (metric invariance), invariant intercepts (scalar invariance), and finally invariant factor covariances (structural invariance). Invariance was assumed to hold if these constrained models fit the data well and if there was minimal difference in their fit from that of the baseline model (Chen, 2007; Wang & Wang, 2020). In line with recommendations by Vandenberg and Lance (2000), the relative fit of constrained models compared to the baseline model was evaluated using change in CFI, RMSEA, and SRMR values, with minor differences of ≤0.02 indicating measurement invariance.

## 3. Results

### 3.1. Factorial Validity

The results from the correlated 4-factor CFA model are presented in Table 1. The 4-factor model showed a good fit to the data overall: CFI = 0.958, SRMR = 0.046, RMSEA = 0.043, 90% CI (0.041, 0.046). The item-to-factor standardized parameter estimates were all significant at *p* < 0.05 and substantively loaded (factor loadings ≥ 0.30), with the exception of two items (9 and 20) that did not substantively load on the Intrapersonal factor. Inter-factor correlations ranged from 0.11 to 0.46 (all were significant at *p* < 0.05).

#### 3.1.1. Measurement Invariance Across Gender and Education Level

For the multi-group CFAs, the configural invariance models for gender and education level showed good fit to the data (Gender: CFI = 0.961, SRMR = 0.051, RMSEA = 0.042, 90% CI [0.039, 0.044]; Education level: CFI = 0.959, SRMR = 0.052, RMSEA = 0.043, 90% CI [0.040, 0.045]), indicating that the item composition of the EQ-i:YV-S scales was equivalent across boys and girls and youth in elementary and high schools. The metric invariance models for gender and education level were comparably well fitting (Gender: CFI = 0.957, SRMR = 0.053, RMSEA = 0.043, 90% CI [0.040, 0.045]; Education level: CFI = 0.954, SRMR = 0.054, RMSEA = 0.044, 90% CI [0.042, 0.046]), indicating EQ-i:YV-S items did not function differently for boys and girls or for youth in elementary and high school (change in the CFI, SRMR, and RMSEA was 0.00 for both gender and education level).

The scalar invariance models were also comparably well fitting (Gender: CFI = 0.957, SRMR = 0.052, RMSEA = 0.042, 90% CI [0.040, 0.045]; Education level: CFI = 0.948, SRMR = 0.054, RMSEA = 0.046, 90% CI [0.044, 0.048]), indicating that the mean differences between boys and girls and between youth in elementary and high school capture all mean differences in the shared variance of the EQ-i:YV-S items (change in the CFI, SRMR, and RMSEA was 0.00 for both gender and education level). Finally, the structural invariance models similarly fit the data comparably well (Gender: CFI = 0.954, SRMR = 0.053, RMSEA = 0.043, 90% CI [0.041, 0.046]; Education level: CFI = 0.944, SRMR = 0.056, RMSEA = 0.048, 90% CI [0.045, 0.050]), indicating that there were limited group differences in either the nature of the latent TEI dimensions or the degree of overlap among them (change in the CFI, SRMR, and RMSEA was 0.00 for both gender and education level).

#### 3.1.2. Reliability for the EQ-i:YV-S Scales

Table 2 presents the means, standard deviations, internal reliability (alpha and omega coefficients), and mean inter-item correlations for each of the EQ-i:YV-S scales separately by gender and education level. All alpha and omega coefficients met the criteria for acceptable internal reliability (≥0.70; e.g., Cicchetti, 1994), with the exception of the intrapersonal scale, which was only slightly lower for high school boys. For elementary school boys, the mean inter-item correlations among the EQ-i:YV-S scales ranged from 0.16 to 0.42. For elementary school girls, the mean inter-item correlations ranged from 0.17 to 0.49. For high school boys, mean inter-item correlations ranged from 0.15 to 0.53. For high school girls, mean inter-item correlations ranged from 0.17 to 0.52. For the total sample, mean inter-item correlations ranged from 0.17 to 0.49. Thus, indicating that the scales show good internal reliability.

Table 3 presents 6-month test–retest correlations for each of the EQ-i:YV-S scales, separately by gender. For the total sample, test–retest correlations ranged from moderate to strong: the weakest correlation being for the Interpersonal scale (*r* = 0.41), and the strongest correlation being for the Adaptability scale (*r* = 0.60). The correlations overall for boys and girls also ranged from moderate to large: the weakest correlation being for the Interpersonal scale among girls (*r* = 0.29), and the strongest correlation being for the Adaptability scale among boys (*r* = 0.64).

### 3.2. Group Differences for the EQ-i:YV-S Scales

To assess gender and education level differences across the EQ-i:YV-S scales, a series of gender by education level ANOVAs was conducted. For ANOVAs with the intrapersonal and interpersonal scales, the assumption of homogeneity of variance was violated. However, non-parametric Kruskal–Wallis tests showed identical findings to parametric ANOVAs; thus, for consistency in interpretation, only the results of ANOVAs are presented. All other parametric assumptions were met across the ANOVAs.

For Total EI, there was a significant main effect for education level, *F* (1, 2142) = 17.80, *p* < 0.001, *d* = 0.18, with elementary students scoring slightly higher on Total EI than high school students. There was no significant main effect for gender and no gender by education level interaction. For the intrapersonal scale, there was a significant main effect for gender, *F* (1, 2142) = 5.17, *p* = 0.023, *d* = 0.10, with girls scoring slightly higher than boys on the intrapersonal scale. There was also a significant main effect for education level, *F* (1, 2142) = 4.68, *p* = 0.031, *d* = 0.09, with high school students scoring slightly higher on the intrapersonal scale than elementary school students. There was no significant gender by education level interaction.

For the interpersonal scale, there was a significant main effect for gender, *F* (1, 2142) = 15.36, *p* < 0.001, *d* = 0.17, and a significant main effect for education level, *F* (1, 2142) = 44.55, *p* < 0.001, *d* = 0.29. However, there was also a significant gender by education level interaction, *F* (1, 2142) = 117.36, *p* < 0.001, η^2^ = 0.05. Bonferroni-corrected pairwise comparisons revealed that elementary school boys (*d* = 0.30) and high school girls (*d* = 0.18) had significantly higher interpersonal scores than elementary school girls. However, high school boys showed significantly lower interpersonal scores when compared to all other groups (elementary school boys: *d* = 0.76, elementary school girls: *d* = 0.46, high school girls: *d* = 0.64).

For the adaptability scale, there was no significant main effect for gender. There was a significant main effect for education level, *F* (1, 2142) = 4.23, *p* = 0.040, *d* = 0.09. However, there was also a significant gender by education level interaction, *F* (1, 2142) = 4.35, *p* = 0.037, η^2^ = 0.001. Bonferroni-corrected pairwise comparisons revealed that elementary school girls had slightly higher adaptability scores than high school girls (*d* = 0.18). No other group comparisons were significant for adaptability.

For the stress management scale, there was a significant main effect for gender, *F* (1, 2142) = 3.85, *p* = 0.050, *d* = 0.09, and a significant main effect for education level, *F* (1, 2142) = 23.22, *p* < 0.001, *d* = 0.21. However, there was also a significant gender by education level interaction, *F* (1, 2142) = 4.98, *p* = 0.026, η^2^ = 0.002. Bonferroni-corrected pairwise comparisons showed high school girls had significantly lower stress management scores than all other groups (elementary school boys: *d* = 0.29, high school boys: *d* = 0.18, elementary school girls: *d* = 0.31).

### 3.3. Convergent Validity

To test convergent validity, Table 4 presents Pearson’s correlations between each of the EQ-i:YV-S scales and each of the scales on the TAS-20, separately by gender. Correlations generally ranged from moderate to strong between the EQ-i:YV-S and TAS-20 scales, with the exception of some weak and not significant correlations. For girls, the weakest correlation among the scales was between the EOT and stress management scale (*r* = 0.00), while the strongest correlation was between the DDF and intrapersonal scale (*r* = −0.73). For boys, the weakest correlation among the scales was between the DDF and adaptability scale (*r* = −0.12), while the strongest correlation was between the DDF and intrapersonal scale (*r* = −0.64).

## 4. Discussion

The present study aimed to address current gaps in the psychometric literature on the EQ-i:YV-S by examining whether the measure has a replicable factor structure as a stand-alone measure using a large sample of elementary and high school students ages 9 to 16. Confirmatory factor analysis (CFA) models showed the EQ-i:YV-S has a replicable factor structure with the 4-factor model demonstrating acceptable fit. Additionally, measurement invariance testing further showed that the factor structure of the EQ-i:YV-S is equivalent across boys and girls, as well as across elementary and high school students. Thus, the factor structure of the EQ-i:YV-S held well overall across gender and education level, providing further evidence that the measure is appropriate for both children and adolescents in Canada. The latter finding of measurement invariance across education levels, in particular, is a novel finding in the literature that supports the validity of the EQ-i:YV-S as an appropriate measure of TEI in both older children and adolescents; evidence that is currently absent for other youth TEI measures.

It should be noted that the two negatively keyed items on the Intrapersonal factor did not load substantively (i.e., factor loadings < 0.30). Thus, these item factor loadings may be impacted by issues with interpreting negatively keyed items among youth or the effects of a method factor (i.e., negative scoring factor), issues that are well documented in the broader psychometric literature (Bulut, 2021). However, negatively keyed items on other factors of the EQ-i:YV-S loaded as expected, and prior research suggests that such items have minimal impact on the overall validity of measures in youth (e.g., personality, ESCs). At the same time, these items meaningfully address potential response biases that can compromise unbalanced measures (i.e., those including only positively keyed items; Lindwall et al., 2012; Primi et al., 2020). Nonetheless, future studies examining the replicability of these factor loadings in other English-speaking samples are required before determinations about the need for revisions to the EQ-i:YV-S can be made.

Consistent with our expectations, the pattern of associations among the four TEI latent dimensions (Interpersonal, Intrapersonal, Stress Management, and Adaptability) in the CFA models indicated four relatively orthogonal dimensions (the mean inter-factor correlation was 0.25). These findings provide further evidence for the multidimensional nature of the TEI construct and underscore the importance of brief measures that provide more than just a single, global score for TEI (a feature of other widely used measures in the field like the SREIT and the TEIQue-ASF; Davis & Wigelsworth, 2018). In particular, it is noteworthy that the Stress Management factor consistently showed the weakest correlations with the other three factors; a pattern not seen in English-speaking samples on adult versions of the measure (e.g., the EQ-i short form; Parker et al., 2011). This finding may point to developmentally related differences in the integration of emotional and social competencies among English-speaking youth. Indeed, previous studies have found that emotional awareness and understanding competencies among youth are less integrated with emotion regulation competencies (e.g., modification and acceptance strategies) than among young adults (Braet et al., 2026).

The creation of short forms from a longer assessment tool often comes with reduced internal reliability (Kruyen et al., 2013); however, alpha coefficients in the present study suggest the EQ-i:YV-S scales overall have good internal reliability. Alpha coefficients in the present study ranged from 0.73 to 0.82 for elementary boys, 0.73 to 0.85 for elementary girls, 0.66 to 0.87 for high school boys, and 0.72 to 0.87 for high school girls. This finding is consistent with previous studies on English-speaking samples (Davis & Wigelsworth, 2018), which likewise found good internal reliabilities for the measure.

In addition, consistent with normative data from Bar-On and Parker (2000) and more recent findings by Esnaola et al. (2018) in a Chinese sample, 6-month test–retest correlations for the EQ-i:YV-S in elementary school students showed the measure has good test–retest reliability (the mean test–retest correlation was 0.52 for the total sample). These findings extend results from previous English-speaking samples, which only tested three-week test–retest correlations for the EQ-i:YV-S (Bar-On & Parker, 2000), by demonstrating good test–retest reliability over a longer six-month period. This finding further supports the temporal stability of scores on the EQ-i:YV-S scales over a substantial period of time in elementary school-aged youth, an important feature of any psychological assessment that purports to measure a trait-like construct.

The pattern of age and gender differences varied considerably across the TEI scales, suggesting that developmental and gender-related factors may influence specific components of emotional functioning in distinct ways. On the Intrapersonal scale, girls and older students tended to score higher, indicating that self-awareness and emotional understanding may strengthen with age and be more pronounced among female students. On the Interpersonal scale, a more complex pattern emerged: younger boys and older girls achieved higher scores, whereas older boys demonstrated the lowest scores of any group. These findings may suggest gender differences in the developmental trajectory of interpersonal competencies such as social awareness and empathy. This is consistent with previous studies showing interpersonal competencies tend to increase with age in youth; however, girls are rated as having better interpersonal competencies than boys across elementary school years, with this advantage increasing in early adolescence (Hajovsky et al., 2022; Rimm-Kaufman et al., 2025). However, it should be noted that boys tended to have greater heterogeneity in their interpersonal competency levels than girls (Hajovsky et al., 2022).

For Adaptability, elementary school girls scored slightly higher than secondary school girls, possibly reflecting greater openness and flexibility in younger students that diminishes somewhat during adolescence. Finally, on the Stress Management scale, older girls scored lower than all other groups, indicating potential challenges in emotion regulation and coping strategies during the transition to adolescence. This is consistent with previous research showing self-management and adaptive emotion regulation are poorer in adolescents than in children (Rimm-Kaufman et al., 2025; Zimmermann & Iwanski, 2014). Together, these patterns highlight the nuanced and evolving nature of emotional competencies across developmental stages and between genders.

As expected, the pattern of generally moderate to strong negative correlations in boys and girls between the EQ-i:YV-S and TAS-20 scales further supports the convergent validity of the EQ-i:YV-S as a brief measure of emotional and social competencies in youth. This pattern of relationships between scales on the EQ-i:YV-S and the TAS-20 is consistent with previous research on adults using the TAS-20 and both the original EQ-i (Parker et al., 2001) and the EQ-i short form (EQ-i:S; Parker et al., 2011). Overall, these findings provide good preliminary evidence that the EQ-i:YV-S has appropriate convergent validity, including novel evidence of its convergent validity with the alexithymia construct, which has a long history of overlap with EI in the broader adult literature (Davidson & Morales, 2022; Salovey & Mayer, 1990).

**Strengths, Limitations, and Future Directions**

Despite evidence of good psychometric properties for the EQ-i:YV-S, the psychometric properties of any measure cannot be definitively determined by a single study. As such, our findings from the present study should be considered in light of some limitations. Firstly, our sample included only youth ages 9 to 16. Future studies would therefore benefit from using a sample of youth at least as young as 7 years old (i.e., the minimum age for which the EQ-i:YV-S was designed) and youth older than 16. Additionally, we were unable to examine ethnic or cultural group differences among the youth in our present sample. Thus, future research would benefit from collecting more detailed data on the ethnic and cultural backgrounds of participants and ideally conducting analyses to test for any group differences in the psychometric properties of the EQ-i:YV-S across these groups (e.g., cross-cultural measurement invariance). This future work will be important to examine the generalizability of our findings to other ethnic and cultural groups. It should also be noted that our analysis of the test–retest reliability of the EQ-i:YV-S was limited to a subset (*n* = 133) of our total sample, specifically of elementary school students. This limits conclusions regarding the temporal stability of the EQ-i:YV-S scales in older adolescents, with future studies needed to examine whether the stability of the measure found for older children and young adolescents persists in older adolescents.

Finally, our analysis of convergent validity was limited to the alexithymia construct, assessed through a self-report measure and only in elementary school students, and we did not conduct any direct tests of discriminant or predictive validity. The issue of discriminant validity is particularly noteworthy as youth TEI measures have rarely been assessed for discriminant validity (Esnaola et al., 2018; Nguyen et al., 2025), unlike their adult measure counterparts (Di Fabio et al., 2016), limiting conclusions regarding the inclusion of construct-irrelevant variance on youth TEI measures. Future studies are, therefore, needed to further examine the convergent, discriminant, and predictive validity of the EQ-i:YV-S with measures of other theoretically related (e.g., five-factor personality traits) and unrelated constructs (e.g., cognitive ability), and important life outcomes (e.g., mental health symptoms, well-being), particularly through a multi-method approach and in multiple education levels.

**Practical Implications**

Nonetheless, the findings of the present study add to the limited literature on the psychometric properties of the EQ-i:YV-S. They provide further evidence that this brief, youth TEI measure demonstrates a replicable factor structure across genders and education levels, good internal consistency and test–retest reliability, and adequate convergent validity in English-speaking Canadian youth. In particular, these findings provide further evidence that the EQ-i:YV-S may be an appropriate tool in educational and clinical contexts where other, lengthier, and/or unidimensional TEI measures designed for youth may be less efficient and provide less dimensional information. However, further research on the psychometric properties of the EQ-i:YV-S is needed.

Moreover, the findings provide further evidence for general and gender-specific developmental trajectories of ESCs and point to the need for tailored programming in educational and clinical settings. For instance, the relative independence of stress management from the other factors on the EQ-i:YV-S points to the need for programming that assists youth with better integrating emotion regulation and coping strategies with other ESCs. More specifically, findings regarding lower stress management and adaptability in older girls suggest the need for targeted programming in high schools to improve girls’ use of adaptive emotion regulation and coping strategies. In contrast, the findings regarding interpersonal competencies in older boys suggest the need for programming in high schools that targets recognizing others’ emotions and interpersonal skills. Educators and clinicians may benefit from considering these general and gender-specific developmental trajectories when designing TEI-based programming for youth.

## Figures and Tables

**Table 1:** Standardized parameter estimates from the four-factor CFA model (*N* = 2146).

Item	INTRA	INTER	ADAPT	STRES
2.	0.70			
5.	0.79			
9.	0.19			
11.	0.78			
16.	0.79			
20.	0.24			
1.		0.57		
3.		0.61		
14.		0.63		
18.		0.57		
22.		0.56		
24.		0.53		
7.			0.65	
10.			0.75	
12.			0.69	
15.			0.72	
17.			0.66	
19.			0.72	
4.				0.50
6.				0.60
8.				0.73
13.				0.81
21.				0.68
23.				0.58
Inter-Factor Correlations				
INTRA		-		
INTER		0.32	-	
ADAPT		0.30	0.46	-
STRES		0.11	0.15	0.18

*Note*. All parameters are significant at *p* < 0.001. INTRA = Intrapersonal; INTER = Interpersonal; ADAPT = Adaptability; STRES = Stress management.

**Table 2:** Means, standard deviations, sample sizes, internal reliability coefficients, and mean inter-item correlations (MIIC) for EQ-i:YV-S, separately by gender and education level.

Scale	Elementary School					High School					Total				
	α	ω	MIIC	N	M (SD)	α	ω	MIIC	N	M (SD)	α	ω	MIIC	N	M (SD)
Intrapersonal Scale															
Boys	0.73	0.74	0.33	582	2.30 (0.62)	0.66	0.69	0.26	473	2.38 (0.56)	0.70	0.72	0.30	1055	2.32 (0.59)
Girls	0.76	0.77	0.36	497	2.38 (0.65)	0.80	0.81	0.41	594	2.40 (0.65)	0.78	0.79	0.39	1091	2.39 (0.65)
Total	0.74	0.76	0.34	1079	2.33 (0.63)	0.75	0.76	0.34	1067	2.39 (0.61)	0.75	0.75	0.34	2147	2.36 (0.62)
Interpersonal Scale															
Boys	0.74	0.74	0.32	582	3.40 (0.49)	0.75	0.75	0.34	473	3.02 (0.56)	0.77	0.78	0.36	1055	3.23 (0.59)
Girls	0.73	0.73	0.32	497	3.25 (0.50)	0.72	0.72	0.30	594	3.34 (0.46)	0.73	0.73	0.31	1091	3.30 (0.48)
Total	0.74	0.74	0.33	1079	3.33 (0.50)	0.76	0.76	0.35	1067	3.20 (0.53)	0.75	0.75	0.34	2147	3.26 (0.52)
Adaptability Scale															
Boys	0.81	0.82	0.42	582	2.74 (0.59)	0.87	0.87	0.53	473	2.74 (0.67)	0.84	0.84	0.47	1055	2.74 (0.63)
Girls	0.85	0.85	0.49	497	2.78 (0.64)	0.87	0.87	0.52	594	2.66 (0.63)	0.86	0.86	0.51	1091	2.71 (0.63)
Total	0.83	0.83	0.45	1079	2.76 (0.61)	0.87	0.87	0.53	1067	2.70 (0.65)	0.85	0.85	0.49	2147	2.73 (0.63)
Stress Management Scale															
Boys	0.81	0.81	0.41	582	2.95 (0.68)	0.79	0.80	0.39	473	2.87 (0.64)	0.80	0.81	0.40	1055	2.92 (0.66)
Girls	0.82	0.83	0.43	497	2.96 (0.67)	0.83	0.83	0.45	594	2.75 (0.68)	0.83	0.83	0.44	1091	2.85 (0.69)
Total	0.81	0.82	0.42	1079	2.95 (0.67)	0.81	0.82	0.42	1067	2.81 (0.67)	0.81	0.82	0.42	2147	2.88 (0.67)
Total Emotional Intelligence Scale															
Boys	0.82	0.81	0.16	582	11.37 (1.54)	0.81	0.76	0.15	473	11.01 (1.50)	0.81	0.78	0.16	1055	11.21 (1.53)
Girls	0.83	0.81	0.17	497	11.36 (1.58)	0.83	0.80	0.17	594	11.52 (1.52)	0.83	0.80	0.17	1091	11.25 (1.55)
Total	0.82	0.81	0.17	1079	11.36 (1.55)	0.82	0.78	0.16	1067	11.09 (1.52)	0.82	0.79	0.17	2147	11.23 (1.54)

*Note.* One participant did not provide their gender and was excluded from calculations for specific gender groups.

**Table 3:** Six-month test–retest correlations for EQ-i:YV-S scales.

Scale	Total Sample	Boys	Girls
TOTAL EI	0.54 *	0.54 *	0.54 *
INTRA	0.54 *	0.60 *	0.46 *
INTER	0.41 *	0.37 *	0.29 *
ADAPT	0.60 *	0.64 *	0.57 *
STRESS	0.52 *	0.52 *	0.52 *

*Note.* TOTAL EI = Total EQ-i:YV-S scale; INTRA = Intrapersonal scale; INTER = Interpersonal scale; ADAPT = Adaptability scale; STRESS = Stress management scale. * *p* < 0.05.

**Table 4:** Correlations between scales on the EQ-i:YV-S and TAS-20 in boys and girls.

Scale	TAS-20	DIF	DDF	EOT
TOTAL EI				
Boys	−0.64 *	−0.43 *	−0.49 *	−0.43 *
Girls	−0.64 *	−0.48 *	−0.53 *	−0.45 *
Total	−0.64 *	−0.45 *	−0.51 *	−0.44 *
INTRA				
Boys	−0.63 *	−0.26	−0.64 *	−0.46 *
Girls	−0.71 *	−0.43 *	−0.73 *	−0.48 *
Total	−0.68 *	−0.36 *	−0.69 *	−0.47 *
INTER				
Boys	−0.36 *	−0.41 *	−0.17	−0.18
Girls	−0.48 *	−0.35 *	−0.46 *	−0.29 *
Total	−0.43 *	−0.35 *	−0.32 *	−0.29 *
ADAPT				
Boys	−0.35 *	−0.24	−0.12	−0.36 *
Girls	−0.37 *	−0.20	−0.19	−0.45 *
Total	−0.34 *	−0.22 *	−0.15	−0.36 *
STRESS				
Boys	−0.51 *	−0.33 *	−0.49 *	−0.27
Girls	−0.21	−0.34 *	−0.11	0.00
Total	−0.34 *	−0.34 *	−0.29 *	−0.13

*Note*. TOTAL EI = Total EQ-i:YV-S scale; INTRA = Intrapersonal scale; INTER = Interpersonal scale; ADAPT = Adaptability scale; STRESS = Stress management scale; TAS-20 = Total Alexithymia; DIF = Difficulty identifying feelings; DDF = Difficulty describing feelings; EOT = Externally oriented thinking. * *p* < 0.05.

## Data Availability

Data are contained within the article.

## References

[B1-ejihpe-16-00081] Ang W. H. D., Lau Y. (2024). Trait emotional intelligence as a predictor of resilience among undergraduate nursing students: A structural equation modelling approach. Nurse Education Today.

[B2-ejihpe-16-00081] Ashkanasy N. M., Daus C. S. (2005). Rumors of the death of emotional intelligence in organizational behavior are vastly exaggerated. Journal of Organizational Behavior.

[B3-ejihpe-16-00081] Bagby R. M., Parker J. D. A., Taylor G. J. (1994). The twenty-item Toronto alexithymia scale—I. Item selection and cross-validation of the factor structure. Journal of Psychosomatic Research.

[B4-ejihpe-16-00081] Bagby R. M., Parker J. D. A., Taylor G. J. (2020). Twenty-five years with the 20-item Toronto alexithymia scale. Journal of Psychosomatic Research.

[B5-ejihpe-16-00081] Bar-On R. (1997). Bar-on emotional quotient inventory: Technical manual.

[B6-ejihpe-16-00081] Bar-On R., Parker J. D. A. (2000). The bar-on EQ-i:YV: Technical manual.

[B7-ejihpe-16-00081] Braet J., Wante L., Keulen J., Bodden D., Vervoort L., Hoorelbeke K. (2026). Age-related differences in emotion regulation: Examining emotion regulation skill interactions in adolescents and young adults. Journal of Youth and Adolescence.

[B8-ejihpe-16-00081] Brown T. A. (2006). Confirmatory factor analysis for applied research.

[B9-ejihpe-16-00081] Bulut H. C. (2021). Item wording effects in psychological measures: Do early literacy skills matter?. Eğitimde ve Psikolojide Ölçme ve Değerlendirme Dergisi.

[B10-ejihpe-16-00081] Cao X., Chen J. (2025). The association between emotional intelligence and prosocial behaviors in children and adolescents: A systematic review and meta-analysis. Journal of Youth and Adolescence.

[B11-ejihpe-16-00081] Caruso D. (2004). Defining the inkblot called emotional intelligence. White paper prepared for the consortium for research on emotional intelligence in organisations.

[B12-ejihpe-16-00081] Chamizo-Nieto M. T., Arrivillaga C., Rey L., Extremera N. (2021). The role of emotional intelligence, the teacher-student relationship, and flourishing on academic performance in adolescents: A moderated mediation study. Frontiers in Psychology.

[B13-ejihpe-16-00081] Chen F. F. (2007). Sensitivity of goodness of fit indexes to lack of measurement invariance. Structural Equation Modeling: A Multidisciplinary Journal.

[B14-ejihpe-16-00081] Cicchetti D. V. (1994). Guidelines, criteria, and rules of thumb for evaluating normed and standardized assessment instruments in psychology. Psychological Assessment.

[B15-ejihpe-16-00081] Davidson D., Morales D. (2022). Associations between autism symptomatology, alexithymia, trait emotional intelligence, and adjustment to college. Frontiers in Psychology.

[B16-ejihpe-16-00081] Davis S. K., Wigelsworth M. (2018). Structural and predictive properties of the emotional quotient inventory youth version–short form (EQ-i:YV-S). Journal of Personality Assessment.

[B17-ejihpe-16-00081] Di Fabio A., Kenny M. E. (2015). The contributions of emotional intelligence and social support for adaptive career progress among Italian youth. Journal of Career Development.

[B18-ejihpe-16-00081] Di Fabio A., Saklofske D. H., Tremblay P. F. (2016). Psychometric properties of the Italian trait emotional intelligence questionnaire (I-TEIQue). Personality and Individual Differences.

[B19-ejihpe-16-00081] Droppert K., Downey L., Lomas J., Bunnett E. R., Simmons N., Wheaton A., Nield C., Stough C. (2019). Differentiating the contributions of emotional intelligence and resilience on adolescent male scholastic performance. Personality and Individual Differences.

[B20-ejihpe-16-00081] Durlak J. A., Mahoney J. L., Boyle A. E. (2022). What we know, and what we need to find out about universal, school-based social and emotional learning programs for children and adolescents: A review of meta-analyses and directions for future research. Psychological Bulletin.

[B21-ejihpe-16-00081] Esnaola I., Arias V. B., Freeman J., Wang Y., Arias B. (2018). Validity evidence based on internal structure of scores of the emotional quotient inventory: Youth version short (EQ-i: YV-S) in a Chinese sample. Journal of Psychoeducational Assessment.

[B22-ejihpe-16-00081] Esnaola I., Freeman J., Sarasa M., Fernández-Zabala A., Axpe I. (2016). Validity evidence based on internal structure of scores of the emotional quotient-inventory: Youth version short (EQ-i:YV-S) in a Spanish sample. Spanish Journal of Psychology.

[B23-ejihpe-16-00081] Feher A., Yan G., Saklofske D. H., Plouffe R. A., Gao Y. (2019). An investigation of the psychometric properties of the Chinese trait emotional intelligence questionnaire short form (Chinese TEIQue-SF). Frontiers in Psychology.

[B24-ejihpe-16-00081] Hajovsky D. B., Caemmerer J. M., Mason B. A. (2022). Gender differences in children’s social skills growth trajectories. Applied Developmental Science.

[B25-ejihpe-16-00081] JASP Team (2024). JASP *(Version 0.19.3) [Computer software]*.

[B26-ejihpe-16-00081] Kruyen P. M., Emons W. H. M., Sijtsma K. (2013). On the shortcomings of shortened tests: A literature review. International Journal of Testing.

[B27-ejihpe-16-00081] Kun B., Urbán R., Paksi B., Csóbor L. V., Oláh A., Demetrovics Z. (2012). Psychometric characteristics of the emotional quotient inventory, youth version, short form, in Hungarian high school students. Psychological Assessment.

[B28-ejihpe-16-00081] Lindwall M., Barkoukis V., Grano C., Lucidi F., Raudsepp L., Liukkonen J., Thøgersen-Ntoumani C. (2012). Method effects: The problem with negatively versus positively keyed items. Journal of Personality Assessment.

[B29-ejihpe-16-00081] Llamas-Díaz D., Cabello R., Megías-Robles A., Fernández-Berrocal P. (2022). Systematic review and meta-analysis: The association between emotional intelligence and subjective well-being in adolescents. Journal of Adolescence.

[B30-ejihpe-16-00081] MacCann C., Jiang Y., Brown L. E. R., Double K. S., Bucich M., Minbashian A. (2020). Emotional intelligence predicts academic performance: A meta-analysis. Psychological Bulletin.

[B31-ejihpe-16-00081] Marsh H. W., Hau K. T., Wen Z. (2004). In search of golden rules: Comment on hypothesis-testing approaches to setting cutoff values for fit indexes and dangers in overgeneralizing Hu and Bentler’s (1999) findings. Structural Equation Modeling.

[B32-ejihpe-16-00081] Mavroveli S., Sánchez-Ruiz M. J. (2011). Trait emotional intelligence influences on academic achievement and school behaviour. British Journal of Educational Psychology.

[B33-ejihpe-16-00081] McElravy L. J., Hastings L. J. (2014). Profiling the youth leader: Personality and emotional intelligence trends and their relationship to leadership skills. Journal of Agricultural Education.

[B34-ejihpe-16-00081] Navarro-Roldán C. P., Mateus-Gómez S., Botero Ruge M. C., Velez G. (2023). Validity and reliability of Spanish version of the EQ-i: YV[S] in Colombian children and youth. International Journal of Psychological Research.

[B35-ejihpe-16-00081] Nguyen Q.-A. N., Tran T., Tran T.-A., Nguyen T.-V., Fisher J. (2025). Validation of the trait emotional intelligence questionnaire—Adolescent short form (TEIQue-ASF) among adolescents in Vietnam. SSM-Mental Health.

[B36-ejihpe-16-00081] Özal Z., Ambrosini F., Biolcati R., Trombini E., Mavroveli S., Mancini G. (2024). Exploring emotional intelligence in children using the trait emotional intelligence questionnaire: A systematic review. BMC Psychology.

[B37-ejihpe-16-00081] Palmer B., Stough C. (2001). Workplace SUIET: Swinburne University emotional intelligence test-descriptive report.

[B38-ejihpe-16-00081] Parker J. D. A., Keefer K. V., Wood L. M. (2011). Toward a brief multidimensional assessment of emotional intelligence: Psychometric properties of the emotional quotient inventory—Short form. Psychological Assessment.

[B39-ejihpe-16-00081] Parker J. D. A., Saklofske D. H., Keefer K. V. (2017). Giftedness and academic success in college and university. Gifted Education International.

[B40-ejihpe-16-00081] Parker J. D. A., Saklofske D. H., Wood L. M., Collin T., Stough C., Saklofske D. H., Parker J. D. A. (2009). The role of emotional intelligence in education. Assessing emotional intelligence: Theory, research, and applications.

[B41-ejihpe-16-00081] Parker J. D. A., Summerfeldt L. J., Walmsley C., O’Byrne R., Dave H. P., Crane A. G. (2021). Trait emotional intelligence and interpersonal relationships: Results from a 15-year longitudinal study. Personality and Individual Differences.

[B42-ejihpe-16-00081] Parker J. D. A., Taylor G. J., Bagby R. M. (2001). The relationship between emotional intelligence and alexithymia. Personality and Individual Differences.

[B43-ejihpe-16-00081] Petrides K. V., Furnham A. (2001). Trait emotional intelligence: Psychometric investigation with reference to established trait taxonomies. European Journal of Personality.

[B44-ejihpe-16-00081] Petrides K. V., Pita R., Kokkinaki F. (2007). The location of trait emotional intelligence in personality factor space. British Journal of Psychology.

[B45-ejihpe-16-00081] Primi R., de Fruyt F., Santos D., Antonoplis S., John O. P. (2020). True or false? Keying direction and acquiescence influence the validity of socio-emotional skills items in predicting high school achievement. International Journal of Testing.

[B46-ejihpe-16-00081] Rimm-Kaufman S. E., Soland J., Kuhfeld M. (2025). Social and emotional competency development from fourth to 12th grade: Relations to parental education and gender. American Psychologist.

[B47-ejihpe-16-00081] Salovey P., Mayer J. D. (1990). Emotional intelligence. Imagination, Cognition, and Personality.

[B48-ejihpe-16-00081] Schutte N. S., Malouff J. M., Hall L. E., Haggerty D. J., Cooper J. T., Golden C. J., Dornheim L. (1998). Development and validation of a measure of emotional intelligence. Personality and Individual Differences.

[B49-ejihpe-16-00081] Siegling A. B., Saklofske D. H., Petrides K. V., Boyle G. J., Saklofske D. H., Matthews G. (2015). Measures of ability and trait emotional intelligence. Measures of personality and social psychological constructs.

[B50-ejihpe-16-00081] Storey-Hurtubise E., Forristal J., Henning C., Parker J. D. A. (2022). Developing emotional and social competencies in children: Evaluating the impact of a classroom-based program. Canadian Journal of School Psychology.

[B51-ejihpe-16-00081] Taibolatov K. M., Pfeyfer N. E., Burdina E. I., Kudysheva A. A., Bolatov A. K. (2024). The role of emotional intelligence on academic motivation of schoolchildren. Frontiers in Education.

[B52-ejihpe-16-00081] Taylor R., Oberle E., Durlak J. A., Weissberg R. P. (2017). Promoting positive youth development through school-based social and emotional learning interventions: A meta-analysis of follow-up effects. Child Development.

[B53-ejihpe-16-00081] Vandenberg R., Lance C. E. (2000). A review and synthesis of the measurement invariance literature: Suggestions, practices, and recommendations for organizational research. Organizational Research Methods.

[B54-ejihpe-16-00081] Van Rens S. M., Henning C. T., Crane A. G., Parker J. D. A. (2024). Trait emotional intelligence revisited: Development and validation of a short measure for personal intelligence. Personality and Individual Differences.

[B55-ejihpe-16-00081] Vega A., Cabello R., Megías-Robles A., Gómez-Leal R., Fernández-Berrocal P. (2022). Emotional intelligence and aggressive behaviors in adolescents: A systematic review and meta-analysis. Trauma, Violence, & Abuse.

[B56-ejihpe-16-00081] Wang J., Wang X. (2020). Structural equation modeling: Applications using Mplus.

[B57-ejihpe-16-00081] Wong C. S., Law K. S. (2002). Development of an emotional intelligence instrument and an investigation of its relationship with leader and follower performance and attitudes. Leadership Quarterly.

[B58-ejihpe-16-00081] Zeidner M., Matthews G., Roberts R. D. (2012). The emotional intelligence, health, and well-being nexus: What have we learned and what have we missed?. Applied Psychology: Health and Well-Being.

[B59-ejihpe-16-00081] Zimmermann P., Iwanski A. (2014). Emotion regulation from early adolescence to emerging adulthood and middle adulthood: Age differences, gender differences, and emotion-specific developmental variations. International Journal of Behavioral Development.

